# Extracellular Vesicles and Host–Pathogen Interactions: A Review of Inter-Kingdom Signaling by Small Noncoding RNA

**DOI:** 10.3390/genes12071010

**Published:** 2021-06-30

**Authors:** Bruce A. Stanton

**Affiliations:** Department of Microbiology and Immunology, Geisel School of Medicine at Dartmouth, Hanover, NH 03755, USA; Bruce.A.Stanton@Dartmouth.edu

**Keywords:** outer membrane vesicles (OMV), extracellular vesicles (EVs), bacterial extracellular vesicles (BEV), small non-coding RNA (sRNA), inter-kingdom communication

## Abstract

The focus of this brief review is to describe the role of noncoding regulatory RNAs, including short RNAs (sRNA), transfer RNA (tRNA) fragments and microRNAs (miRNA) secreted in extracellular vesicles (EVs), in inter-kingdom communication between bacteria and mammalian (human) host cells. Bacteria secrete vesicles that contain noncoding regulatory RNAs, and recent studies have shown that the bacterial vesicles fuse with and deliver regulatory RNAs to host cells, and similar to eukaryotic miRNAs, regulatory RNAs modulate the host immune response to infection. Recent studies have also demonstrated that mammalian cells secrete EVs containing miRNAs that regulate the gut microbiome, biofilm formation and the bacterial response to antibiotics. Thus, as evidence accumulates it is becoming clear that the secretion of noncoding regulatory RNAs and miRNAs in extracellular vesicles is an important mechanism of bidirectional communication between bacteria and mammalian (human) host cells. However, additional research is necessary to elucidate how noncoding regulatory RNAs and miRNA secreted in extracellular vesicles mediate inter-kingdom communication.

## 1. Introduction

Numerous studies have demonstrated that extracellular vesicles (EVs) play an important role in cell–cell signaling in all three domains of life, and that EVs are an important mechanism of intra-kingdom and inter-kingdom communication, including communication between hosts and pathogens (reviewed in [1,2,3,4,5,6,7,8,9,10,11,12,13]). The secretion of EVs as a mechanism of intra-kingdom and inter-kingdom communication is evolutionarily conserved, as cells from prokaryotes, plants, and animals release EVs [14,15,16]. Intercellular communication mediated by EVs is an important aspect of host–pathogen interaction that does not require direct cell–cell contact, since, for example, many bacteria reside in mucus overlying epithelial cells in the gut and lungs [17,18]. EVs are spherical, cell-derived fragments ~10 nm to 1000 nm in diameter delimited by a lipid bilayer and contain proteins, lipids, nucleic acids and many other cellular constituents [2,8,19,20,21]. The lipid bilayer protects intra-vesicular RNA from extra-vesicular RNases, and proteins from extracellular proteases, thereby increasing the biological half-life of the intra-vesicular content [18,22]. However, studies have shown that RNA and proteins also decorate the extra-vesicular surface of EVs and have biological activity [23]. EVs have been identified in all biological fluids and organs [24], have long half-lives in blood (30 min to 6 h [25,26]), enter cells by a variety of mechanisms, and once inside target cells, release their contents whereupon proteins, small RNAs (sRNAs), tRNA fragments and microRNAs (miRNAs) regulate recipient cell gene expression and function [1,2,3,4,5,6,7,8,9,10,11,12,13]. Currently, eukaryotic EVs are classified into many subgroups, including exosomes, microvesicles, and apoptotic bodies, based on their origin, biogenesis, size (~10 to 1000 nm) and composition [10,21,27,28]. However, at the present time there is no consensus on EV subtypes due to a variety of issues, including the cellular source, overlap in size, composition, the lack of knowledge about their biogenesis, and difficultly in isolating specific subtypes [10,27]. The International Society of Extracellular Vesicles recommends the use of the term EVs in publications since most studies do not identify the subtype [21,27,28,29]. Thus, in this review, the term EVs will be used as a general term to describe vesicles secreted by non-microbial cells. The term outer membrane vesicle (OMV) will be used to describe EVs secreted by Gram-negative bacteria. OMVs are ~10 to 300 nm vesicles delimited by the outer membrane of Gram-negative bacteria and their contents include periplasmic and cytoplasmic components, including sRNA, tRNA fragments, DNA, lipids and proteins [18,30,31,32,33,34,35,36,37]. Gram-negative bacteria also secrete outer-inner membrane vesicles (O-IMV), which are delimited by the outer and inner membrane of the bacteria, although secretion of O-IMV represents less that 1% of the vesicles secreted by Gram-negative bacteria [38,39]. The role of O-IMV in interdomain signaling is not clear [38,39]. Gram-positive bacteria also secrete EVs, but since Gram-positive bacteria do not contain an outer membrane, these EVs are often described as bacterial extracellular vesicles (bEV or BEV) [10,19,20,40,41,42,43,44,45,46]. In this review, the terms EVs, OMVs and BEVs will be used to describe EVs secreted by non-microbial cells, Gram-negative bacteria, and Gram-positive bacteria, respectively. Many recent studies have shown that OMVs and BEVs secreted by bacteria deliver sRNA and tRNA fragments (~18 to 50 nucleotides (nt) long) to mammalian (human) cells and, although many details are lacking, it has been suggested that the sRNA and tRNA fragments regulate target cell gene expression by sequestering regulatory proteins and/or by base pairing with target mRNAs [4,18,22,30,31,32,41,47,48,49,50,51,52,53,54,55,56]. sRNAs are heterogeneous in size (~20 to 500 nt) and regulate gene expression by base-pairing with the translation initiation region or coding sequence of target mRNAs or by acting as sRNA sponges, which are produced by transgenes and have complementary binding sites to specific miRNAs [13]. tRNAs are ~70 to 100 nt long molecules with highly conserved sequences that form secondary cloverleaf and L-shaped three-dimensional structures [13,57,58,59]. tRNA fragments have been shown to mediate gene silencing in eukaryotic cells through an argonaute (Ago2)-microRNA-like mechanism and have both negative and positive effects on protein translation [57,58,59]. Many excellent and comprehensive reviews on EVs, OMVs and BEVs have been published [1,2,3,4,5,6,7,8,9,10,11,12,13]. However, very few studies have been published describing the role of extracellular vesicles as a delivery mechanism for sRNA, tRNA fragments and miRNAs to target cells and elucidating the mechanism whereby they mediate inter-kingdom signaling. Thus, in this review, the focus will be on recent studies describing how sRNAs, tRNA fragments and miRNAs secreted inside vesicles mediate host–pathogen interactions between bacteria and mammalian (human) cells. In addition, unresolved issues and research opportunities will be discussed.

## 2. Extracellular Vesicles Secreted by Bacteria Are Important Mediators of Inter-Kingdom Communication and Deliver sRNA and tRNA Fragments to the Mammalian Host

OMVs and BEVs secreted by microbes serve a variety of intra-kingdom functions, including horizontal gene transfer, quorum sensing, biofilm formation, pathogenicity, depredation, antibiotic resistance, defense, and nutrient acquisition [1,2,3,4,5,6,7,8,9,10,11,12,38,60,61]. Similar to miRNAs, every bacterial sRNA and tRNA fragment is predicted to have multiple targets, can regulate genes in cis and trans, modulates transcription and translation, and affects mRNA stability [2]. Although, unlike eukaryotes, bacteria do not have a sRNA-mRNA-induced silencing complex (RISC). The Hfq protein in bacteria stabilizes and presents sRNAs to mRNA by a mechanism that resembles RISC in eukaryotes [2].

**Figure 1:**
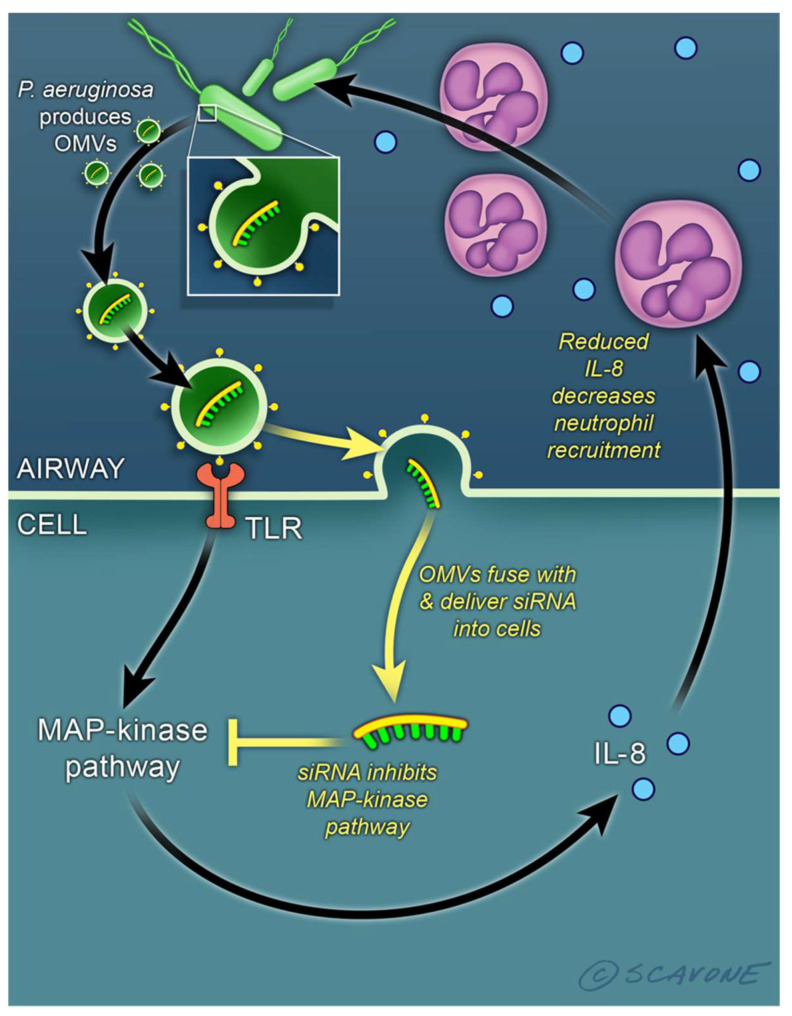
An sRNA (short RNA) secreted in OMVs (outer membrane vesicles) by *P. aeruginosa* suppresses the host immune response. *P. aeruginosa*, residing in mucus overlying lung epithelial cells, secretes OMVs containing sRNA52320, a 23 nt tRNA fragment. OMVs fuse with cholesterol-rich lipid rafts in the apical membrane of airway epithelia cells, and deliver sRNA52320, which reduces the expression of several genes in the LPS stimulated MAPK pathway, thereby reducing IL-8 secretion and the migration of neutrophils into the lungs of mice [18,36,56,62]. Since neutrophils kill *P. aeruginosa,* it is proposed that the secretion of sRNA52320 in OMVs is a mechanism to reduce neutrophil mediated killing of *P. aeruginosa,* thereby enabling *P. aeruginosa* to establish/maintain chronic infection in immunocompromised individuals.

OMVs and BEVs mediate the inter-kingdom transfer of sRNA and tRNA fragments between bacteria and mammalian cells without requiring direct contact [4,30,31,32,41,47,48,49,50,51,52,53,54,55,56]. For example, *Pseudomonas aeruginosa*, residing in the mucus layer overlying lung epithelial cells, secretes OMVs that diffuse through the mucus, fuse with lipid rafts in the apical membrane of airway epithelial cells and deliver a 23 nt tRNA fragment (sRNA-52320) into host cells. sRNA-52320 suppresses the immune response to bacterial infection by targeting several genes in the LPS stimulated MAPK signaling pathway, thereby reducing IL-8 secretion and the migration of neutrophils into the lungs of mice (Figure 1) [18,36,56,62]. Thus, sRNA-52320 is predicted to facilitate the ability of *P. aeruginosa* to establish a chronic lung infection in immunocompromised individuals [18]. sRNAs secreted in OMVs by *Aggregatibacter actinomycetemcomitans,* a periodontic pathogen, can cross the blood–brain barrier, and sRNAs in the OMVs stimulate TNF-a production by activating the NF-κB signaling pathways in macrophages, promoting the secretion of pro-inflammatory cytokines in the brain [63,64,65]. sRNAs in OMVs secreted by *Escherichia coli* are transferred into bladder epithelial cells and suppress LPS-induced IL-1a secretion [66]. In *E. coli* OMVs, over 90% of sequence reads are mapped to tRNA fragments, which are differentially packaged [67]. *Listeria monocytogenes* secretes OMVs containing the sRNA rli32 that stimulates IFN-β production in bone marrow derived macrophages, thereby promoting the intracellular growth of the pathogen [68,69]. Two sRNAs (sR-2509025 and sR-989262) secreted by *Helicobacter pylori* in OMVs reduce LPS-stimulated IL-8 secretion by human gastric adenocarcinoma cells in culture [70]. Although the mechanism whereby bacterial-derived sRNAs regulate gene expression in eukaryotic host cells is not well characterized, several studies suggest that prokaryotic sRNAs use Ago2 to enhance binding to mRNA targets [71,72]. For example, the intracellular pathogen *Mycobacterium marinum* infects human THP-1 macrophage cells and secretes a ∼23-nt sRNA that binds to the host RNA-induced silencing complex (RISC) and interferes with gene expression [72]. In addition, *Salmonella* secretes numerous sRNAs into the cytosol of infected human intestinal epithelial cells, and within the host cell the sRNAs are processed into ~22 nt fragments by the Ago2-based non-classical miRNA processing machinery [71]. One sRNA, Sal-1, facilitates the intracellular survival of *Salmonella* in infected cells [71].

BEVs (~20 nm to 400 nm) secreted by Gram-positive bacteria also contain sRNAs (<100 nt, with most in the range of 10 to 40 nt) [10,19,20,40,41,42,43,44,45,46]. Although recent studies have shown that sRNAs in BEVs affect host cell biology, to date no studies have identified specific sRNAs that mediate the host response to BEVs. *Staphylococcus aureus* secretes sRNA and DNA in BEVs that have been implicated in inducing IFN-β mRNA by activation of Toll-like receptors in mouse macrophages [41]. *S. aureus* secrete BEVs that contain immunostimulatory DNA, RNA and peptidoglycan that activate innate immune receptors and induce autophagy in host A549 epithelial cells [40]. Although *Mycobacterium tuberculosis* (M.tb) is not considered a Gram-positive or Gram-negative bacterium, BEVs secreted by M.tb contain RNAs that promote IFN-b secretion upon entry into RAW 264.7 macrophages, which leads to M.tb clearance via activation of the macrophage RIG-I/MAVS signaling pathway [61]. Importantly, combined treatment with M.tb BEVs and antibiotics decreases bacterial load in a mouse model, reducing lung damage compared to treatment with antibiotics alone [61]. Thus, several studies have demonstrated that Gram-positive bacteria regulate the mammalian (human) host immune response by secreting BEVs containing sRNAs.

## 3. Extracellular Vesicles Secreted by Mammalian Cells Are Important Mediators of Intercellular Communication and Delivery of miRNAs to Bacteria

**Figure 2:**
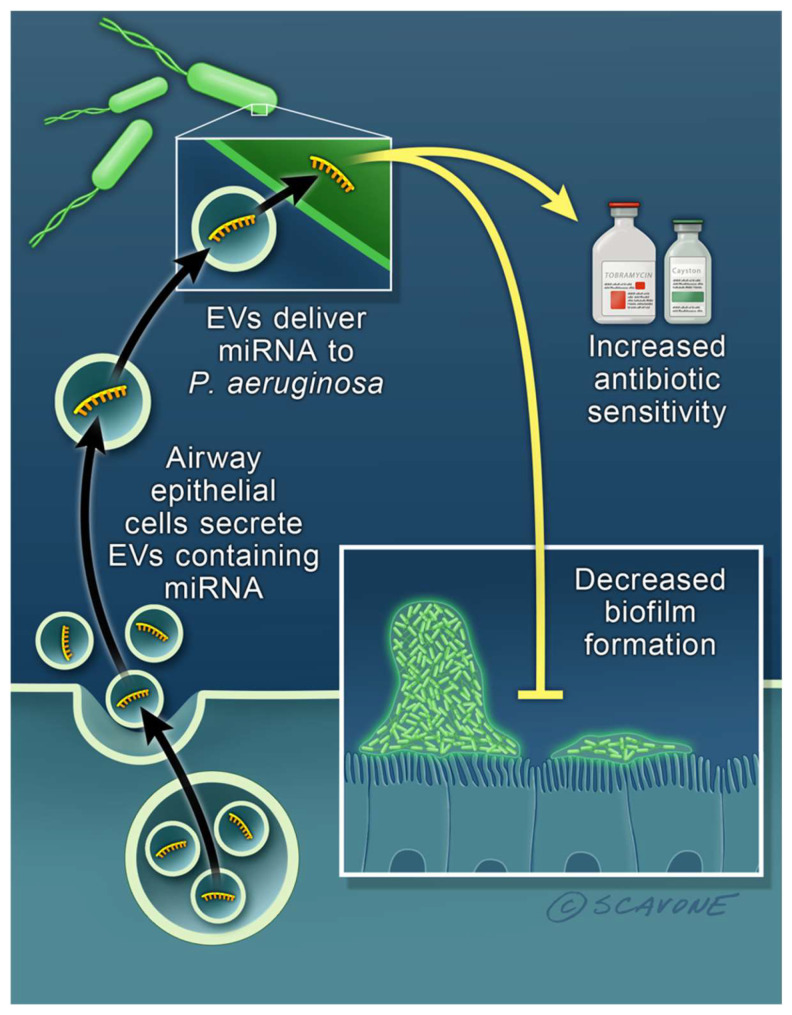
EVs (extracellular vesicles) secreted by mammalian (human) lung epithelial cells contain a miRNA that increase the sensitivity of *P. aeruginosa* to antibiotics and reduces biofilm formation. Airway epithelial cells secrete EVs which deliver the miRNA let-7b-5p to *P. aeruginosa.* Let-7b-5p reduces the formation of antibiotic resistant *P. aeruginosa* biofilms and increases the ability of antibiotics to kill *P. aeruginosa*. A proteomic analysis of *P. aeruginosa* revealed that let-7b-5p increases the ability of β-lactam antibiotics to reduce biofilm formation by decreasing the abundance of several proteins essential for biofilm formation [75].

A few studies have demonstrated that eukaryotic miRNAs (~22 nt long) affect bacterial gene expression and phenotype [10,11,12,27,28]. Transfection of eukaryotic miRNAs into *Fusobacterium nucleatum* and *E. coli* affects mRNA abundance and bacterial growth in the gut [73]. The same research group also found that miR-30d given orally ameliorates autoimmune encephalomyelitis (EAE), a model of multiple sclerosis, by increasing *Akkermansia muciniphila* abundance in the gut, which leads to an increase in regulatory T cells that suppress EAE symptoms [74]. None of these studies, however, examined the ability of EVs to deliver miRNAs to bacterial cells. In a recent study, EVs secreted by primary human airway epithelial cells were shown to deliver the miRNA let-7b-5p to *P. aeruginosa* [75]. Moreover, it was demonstrated that let-7b-5p reduced the formation of antibiotic resistant biofilms by decreasing the abundance of several proteins essential for biofilm formation [75] (Figure 2). The effect of let-7b-5p was inhibited by a let-7b-5p antagomir, which blocked the ability of let-7b-5p to bind to target mRNAs. Let-7b-5p was also predicted to target the corresponding biofilm gene orthologs in *Burkholderia cenocepacia*, another opportunistic lung pathogen, suggesting that the ability of let-7b-5p to reduce biofilm formation and antibiotic sensitivity is not limited to *P. aeruginosa* [75]. Since several let-7 family members are in clinical trials to reduce inflammation and, because chronic *P. aeruginosa* lung infections are associated with a hyper-inflammatory state, treatment with let-7b-5p and a β-lactam antibiotic in nanoparticles or EVs may benefit patients with antibiotic-resistant *P. aeruginosa* infections [75]. Additional studies are required to determine if EVs secreted by other mammalian cells affect bacterial gene expression, how the EVs deliver miRNA to the cytoplasm of bacteria and to elucidate the mechanism whereby a eukaryotic miRNA affects prokaryotic gene expression.

Taken together, the studies described above demonstrate that inter-kingdom communication mediated by sRNA, tRNA fragments and miRNA containing EVs is bi-directional: eukaryotic EVs containing miRNA regulate gene expression in bacteria, and conversely, sRNA and tRNA fragments secreted by OMVs and BEVs affect gene expression and function in eukaryotic cells. However, to date, only one study has shown that EVs secreted by mammalian (human) cells deliver a miRNA that directly targets bacterial genes and affects the bacterial phenotype [75].

## 4. Future Prospects

This brief review has summarized the current state of knowledge regarding our understanding of how small noncoding regulatory RNAs secreted in vesicles mediate inter-kingdom communication between bacteria and mammalian (human) hosts. Several excellent reviews have described how vesicles also mediate signaling between all seven kingdoms of life, including animalia, plantae, and fungi, etc. [2,7,11,12]. However, many aspects of inter-kingdom communication by small regulatory RNAs in vesicles are either poorly understood or unknown and require additional study. For example, the effect of growth conditions, temperature, and antibiotics on vesicle secretion and small regulatory RNA content of OMVs and BEVs are not well understood [22,44,51,76,77]. A few studies have shown that small regulatory RNAs are differentially packaged in vesicles and that the sRNA content of BEVs and OMVs is dependent on growth conditions and antibiotics [22,44,51,76]. Thus, additional studies are required to determine the effect of growth conditions and antibiotics on RNA content of bacterial vesicles, as well as the effect of polymicrobial infections on RNA content of OMVs and BEVs, and how these factors affect the host response to OMVs and BEVs. In addition, the mechanism whereby noncoding regulatory RNAs affect inter-kingdom communication is also not well understood [78]. At least two mechanisms may be used by noncoding regulatory RNAs to mediate inter-kingdom communication [11]. First, RNA molecules secreted by one organism could integrate into the regulatory RNA pathway present in the other. As noted above, RNAs secreted by *Salmonella* and *E. coli* are processed in eukaryotic cells by the RISC complex, but details are unknown [71,79]. Second, some regulatory RNAs are exported in vesicles containing the molecules required for their activity in the recipient cell. For example, Hfq is exported in OMVs secreted by *Yersinia pestis* [76]. Moreover, little is known about the mechanism whereby EVs deliver small regulatory RNAs to bacteria, and very few studies have unambiguously elucidated the specific gene targets and mechanisms whereby small regulatory RNAs affect gene expression in target cells. Additional studies are also required to explore the use of EVs to prevent the formation of multidrug resistant biofilms [75]. The use of EVs as immunotheropeutics and as diagnostic tools for infectious disease also warrant additional research. It should also be noted that vesicles contain other constituents including proteins and virulence factors that mediate host–pathogen interactions [36,80]. Based on presentations at recent international meetings, including the International Society of Extracellular Vesicles 2021 meeting, many of these issues, and more, are being studied. Thus, the future of research in this exciting field of inter-kingdom communication by regulatory RNAs secreted in vesicles is likely to produce many novel insights, which could lead to the development of therapeutics using small regulatory RNAs to treat bacterial infections and reduce bacteria-induced inflammation in the host.
